# Is Eminectomy Effective in the Management of Chronic Closed Lock?

**DOI:** 10.1007/s12663-019-01216-x

**Published:** 2019-04-05

**Authors:** Ketan Shah, Andrew Nicholas Brown, Robert Clark, Mohammed Israr, Donald Starr, Leo F. A. Stassen

**Affiliations:** 1 Morriston Hospital, Swansea, UK; 2 Hull Royal Infirmary, Hull, UK; 3 Department of Oral and Maxillofacial Surgery, University Hospital Bristol, Bristol Royal Infirmary, Upper Maudlin Street, Bristol, BS2 8HW UK; 4 St. James’s Hospital, Dublin, Ireland

**Keywords:** Temporomandibular joint, Temporomandibular joint dysfunction, Closed lock, Eminectomy

## Abstract

**Purpose:**

This study assesses the effectiveness of eminectomy in the management of chronic closed lock, refractory to conservative medical management in the largest multi-centred study of its kind in the UK, with a cohort of 167 patients. Temporomandibular mandibular joint disorder affects 30% of adults in the UK. Chronic closed lock is a well-documented sub-type.

**Method:**

A retrospective study of patients with refractory closed lock was carried out, where conservative management had been implemented for a minimum of 6 months. Refractory patients were offered eminectomy at three separate centres over a period from 1995 to 2011. The primary variable was the inter-incisal distance (IID). Other variables included pain, clicking and nerve damage pre- and post-operatively.

**Results:**

There were 167 patients across all three centres, 81% of which were female. The mean IID was 23 mm pre-operatively and 37 mm post-operatively. There was a statistically significant association with the primary predictor variable, yielding a *p* value of < 0.05. Clicking resolved completely post-operatively in 84 patients (58%). Pain subjectively improved in 56% cases.

**Conclusion:**

Eminectomy is a safe and effective surgical procedure and has a role to play as a second-line surgical option in the management of closed lock after more conservative medical options have failed.

## Introduction

Temporomandibular joint disorder (TMD) is a collective term for a range of different clinical problems that encompass the muscles of mastication, the temporomandibular joint (TMJ) and the associated structures [1, 2]. It is estimated that 20–30% of adults [2, 3] will experience the symptoms of TMD. The aetiology of TMD remains unknown but is thought to include parafunctional activity, stress, previous trauma and internal derangement of the disc complex [2].

Closed lock is a specific type of TMD where there is derangement of the articular disc complex and refers to the displacement of the disc, most commonly anteromedially, without reduction and resultant limited mouth opening [4].

In a normal joint, on initial mouth opening, the condylar head rotates followed by translation along the articular eminence on further opening. The articular disc will translate with the condyle to remain interpositioned between the head and the eminence. In patients with closed lock, the translation of the condyle is limited by the failure of the meniscus to reduce, as a consequence of the disc being displaced, most commonly anteromedially, with the posterior band becoming trapped anterior to the condyle [5]. It has been postulated that the articular disc may be attached to the eminence via adhesions, preventing reduction and has been linked with osteoarthrosis [6].

Closed lock can be described as being acute or chronic depending on the duration of locking [4] and is frequently accompanied with characteristic symptoms including: significant jaw pain, limitations of jaw movement (namely reduced maximal opening) and functional impairment (eating) [7]. However, a number of patients with anteromedial meniscus displacement report no clinic symptoms [8, 9] indicating that other factors are influential to the aetiology and severity of closed lock [9]. These factors include the depth of the glenoid fossa, the steepness of the eminence and the size of the condylar head [8].

The treatment modalities for closed lock are well documented within the literature and include both medical and surgical management options. Medical management includes the use of non-steroidal anti-inflammatories, steroid therapy, rehabilitation with orthotic devices, physical therapy and cognitive behavioural therapy [7]. The minimally invasive surgical options including arthroscopy and arthrocentesis are considered as first-line surgical modalities, with open surgery including: condylar shave, plication procedures and eminectomy of the articular eminence as alternative, secondary surgical procedures [7]. The consensus of the current literature is that surgical treatment for TMJ disc displacement without reduction, including that of closed lock, should be deferred for a minimum of 6 months to allow sufficient time for comprehensive medical management and rehabilitation [4, 7]. Closed lock that proves refractory to non-surgical interventions should then be considered for an interventional surgical approach. In this retrospective study, the rationale for eminectomy as a surgical treatment option for closed lock is the removal of the articular eminence, which thereby eliminates the anatomic structure against which the articular disc becomes trapped on mouth opening and thereby relieves some or all of the symptoms. It has been hypothesised that articular eminectomy may successfully treat patients suffering closed lock and was first described by Stassen and Currie in 1994 [8]. The pilot study concluded that ‘Eminectomy appears to be a safe and simple method of reducing a closed lock of the TMJ’ [8].

Eminectomy as a standard surgical procedure was carried out by a preauricular incision using the Al-Qayat Bramley approach to expose the articular eminence followed by adequate removal and recontouring of the articular eminence using a bone saw [10]. This procedure helps to eliminate mechanical interference and facilitates a smooth functioning surface for the joint translation. Unlike some open TMJ surgical procedures, the procedure is intra-capsular and the disc is not surgically repositioned and there is no interference with the internal joint mechanism [5]. The surgical treatment has a potential risk of damage to the temporal and zygomatic branches of the facial nerve. The literature reports varying degrees of incidence, from 9 to 18% of patients reporting transient weakness of facial nerve post-operatively [4]. The success of the operation can be measured by the following criteria: reduction in pain, improvement in function (mouth opening) and reduction in clicking.

This study aims to assess the effectiveness and role of eminectomy as an alternative surgical option for the management of chronic closed lock.

## Materials and Methodology

Due to the retrospective nature of this study, it was granted an exemption in writing by the University of Leeds IRB.

In this retrospective study, data were collected from three different ‘Oral and Maxillofacial Departments’: Sunderland Royal Infirmary (UK), Hull Royal Infirmary (UK) and St James’ hospital Dublin (Ireland), between the years of 1995 and 2011. All patients who had undergone eminectomy were identified using the operating theatre lists, and the subsequent patient records were requested and analysed. Eminectomies performed only as a treatment for closed lock were included, whilst cases where it had been used for recurrent dislocation were excluded from the study. There were no other exclusion criteria.

The primary outcome variable is the inter-incisal distance at the point of discharge from the clinic at a minimum of 6 months post-operatively. The other outcome variables include pain and clicking improvement at a minimum of 6 months post-operatively.

Wilkes score and visual analogue scores were not recorded for all patients in this retrospective study, and therefore a customised proforma was constructed to facilitate data collection and analysis (Table 1). Using the patients’ medical records, the proforma was completed recording the patient demographics, gender, pre-operative and post-operative symptoms including pain, mouth opening and clicking and any other documented surgical complications.

**Table 1:**
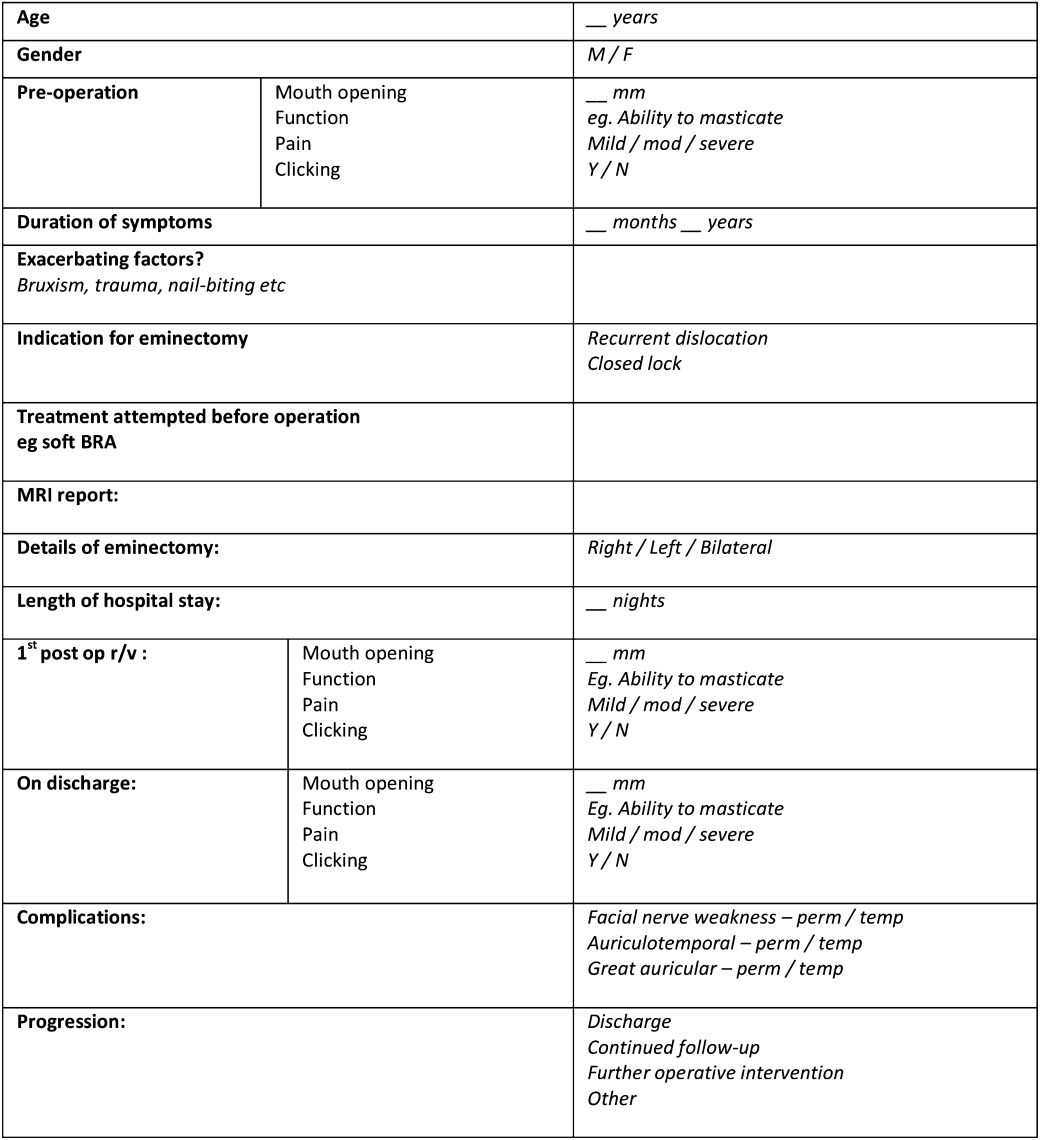
Clinical proforma used in data collection

## Results

Between 1995 and 2011, 167 patients underwent eminectomy for closed lock. The distribution between the three units and gender distribution is shown in Table 2.

**Table 2:** Gender distribution

Hosp/sex	M	F
SRH	9	35
HRI	9	29
Dublin	13	72
Total	31	136
Percentage distribution	19% male	81% Female

**Fig. 1:**
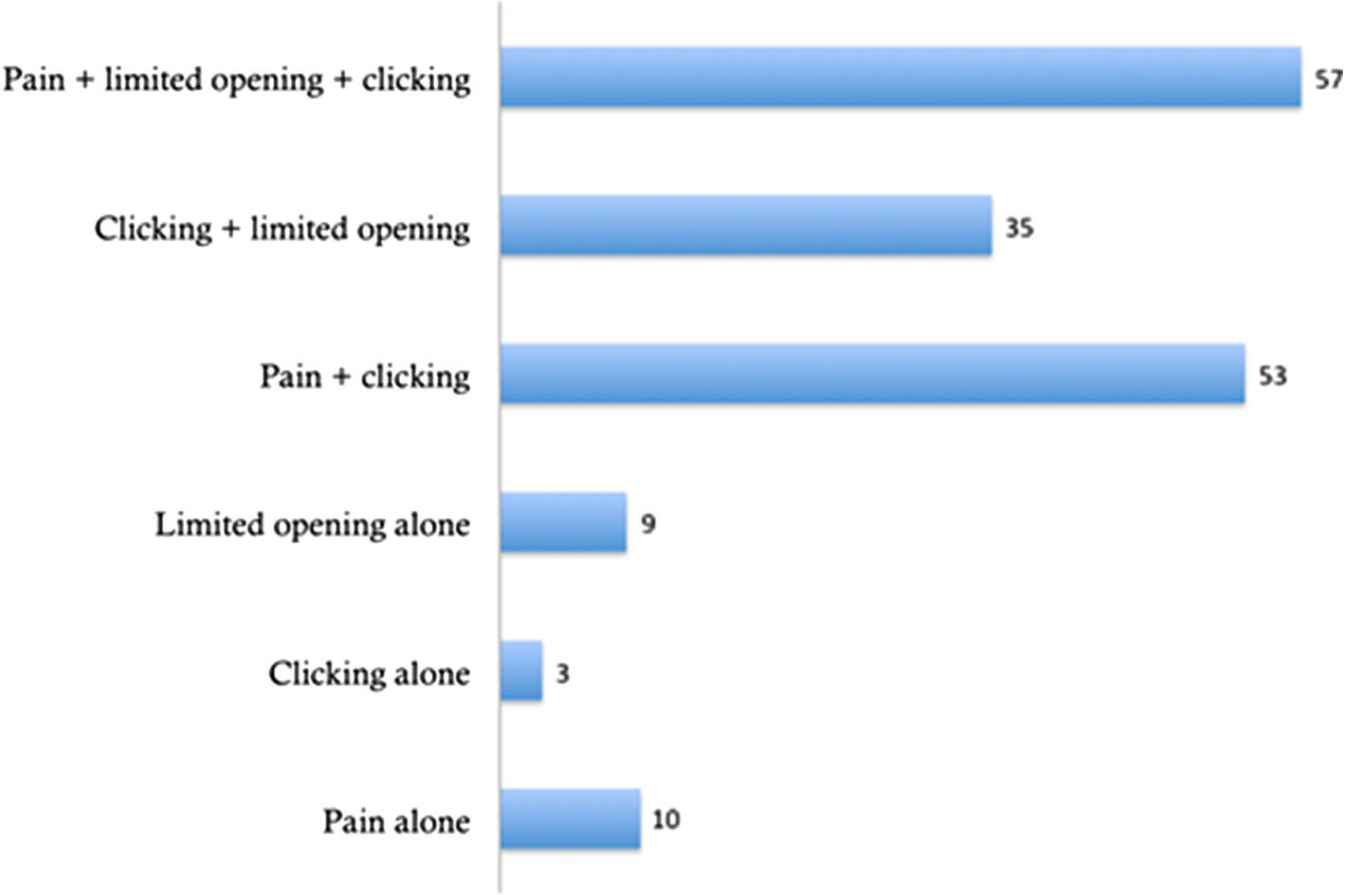
Type and frequency of pre-operative symptoms

Of these patients, 111 underwent a unilateral eminectomy with 56 undergoing a bilateral eminectomy. The mean duration from the onset of symptoms until the initial secondary care consultation was 25 months (range 6 months–20 years) with all patients proving refractory to conservative management, e.g. bite-raising appliances, splints and medications. A total of 102 patients (61%) reported a history of bruxism, mandibular trauma or parafunctional habit, e.g. nail biting. The type and frequency of pre-op symptoms are recorded in Fig. 1. It shows most patients had combined symptoms of either pain and clicking or clicking with limited mouth opening.

**Fig. 2:**
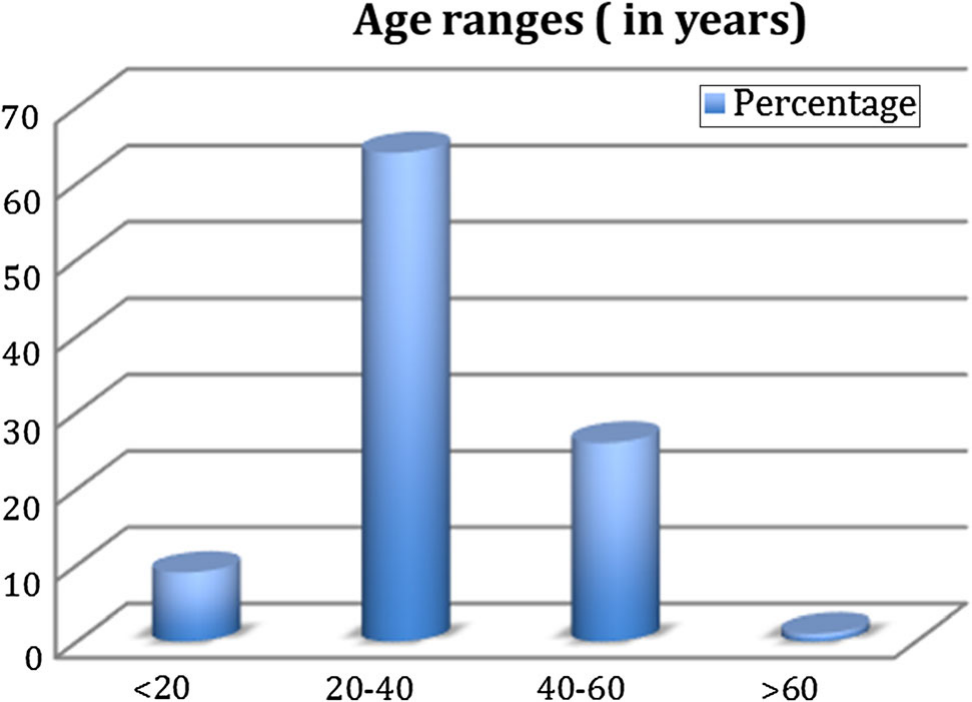
Age distribution of the patient cohort

It is pertinent to note that the majority of patients undergoing eminectomy across the three centres were between 20 and 40 years of age. The age distribution is demonstrated in Fig. 2.

Looking at the three main symptoms of pain, clicking and limited mouth opening and comparing them to the pre-operative symptoms, the results were as follows.

**Fig. 3:**
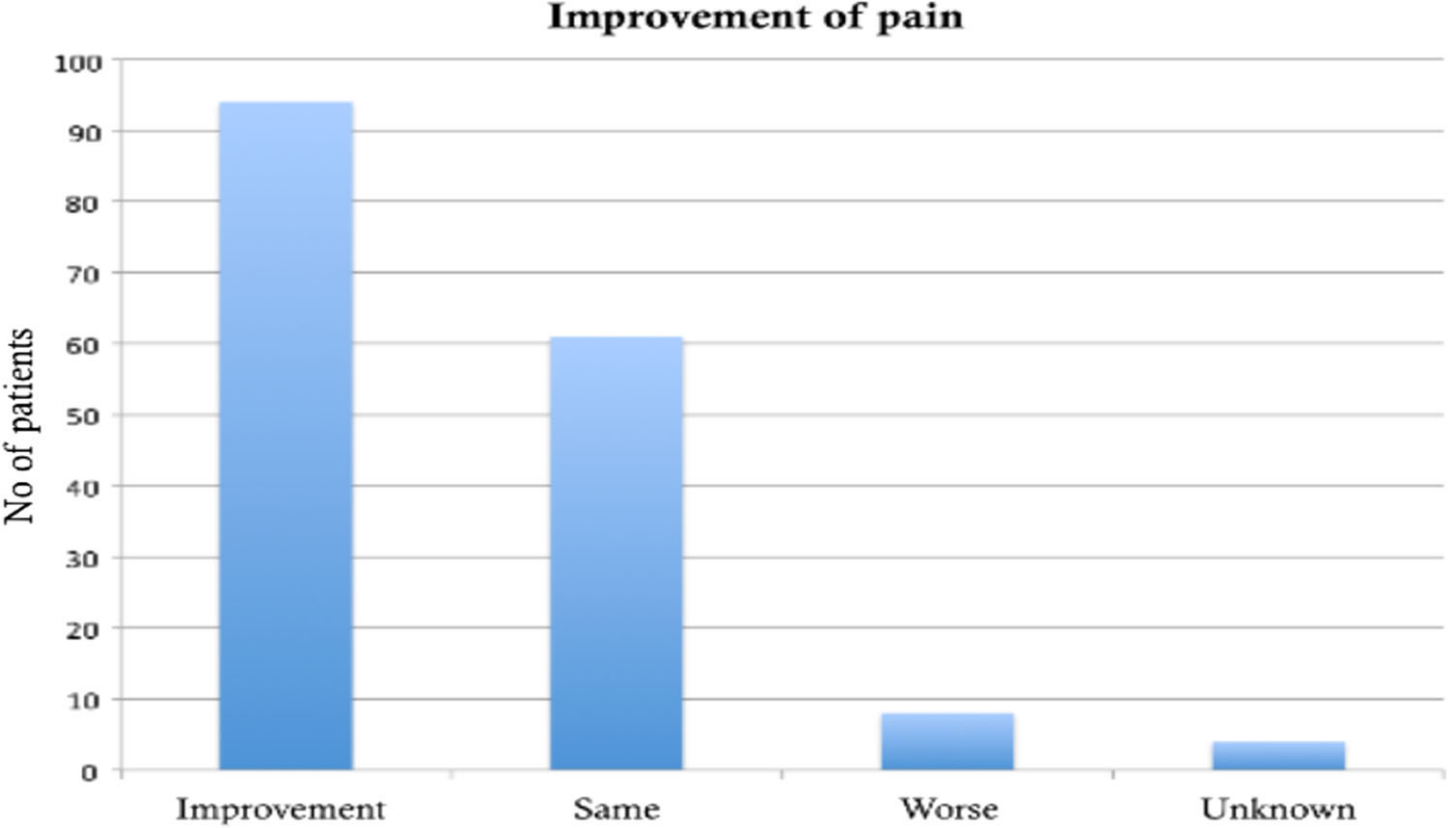
Assessment of pain post-operatively

Pain subjectively improved in 56% cases and remained unchanged in 37% of the cases. It however worsened in 5% of the cohort. This is demonstrated in Fig. 3.

**Fig. 4:**
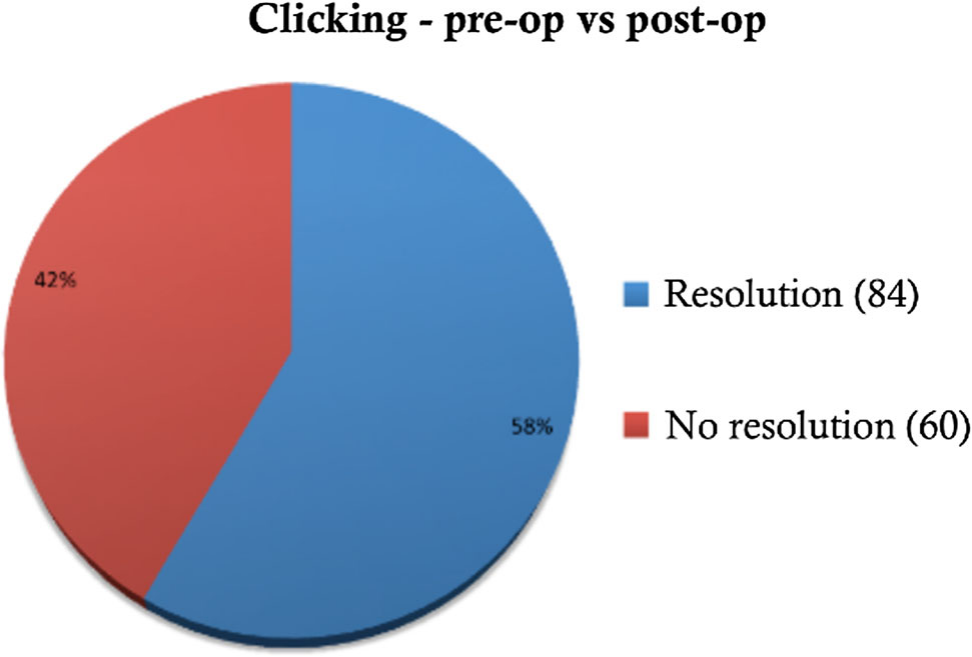
Pre-operative and post-operative clicking

Clicking was present pre-operatively in 144 cases, 11 cases had no clicking, whilst in the remaining 12 cases the notes failed to record the symptom. Post-operatively, the clicking resolved in 84 patients (58%) completely, whilst the remaining 60 cases (42%) continued to have clicking after surgery. This is highlighted in Fig. 4.

**Fig. 5:**
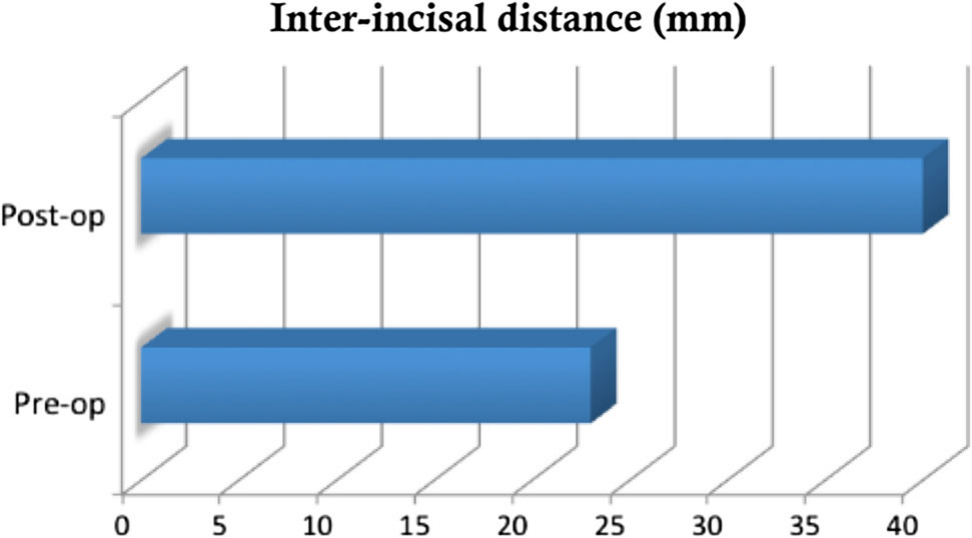
Inter-incisal distance (IID) pre- and post-operatively

In 159 out of 167 cases, it was possible to assess improvements in mouth opening, by comparing the inter-incisal distance (IID) pre-operatively and at discharge. The mean pre-operative IID was 23 mm, whereas the mean IID at discharge was 37 mm. This is exhibited in Fig. 5. Inter-incisal distance is the primary treatment outcome marker for determining the success of eminectomy as a surgical procedure for the correction of a closed lock. Table 3 highlights the pre-operative and post-operative IIDs across all three centres. The data in black represent the data from Dublin, those in red represent the data from Hull, and those in blue represent the data from Sunderland. A paired ‘*t’* test was performed yielding a *p* value of < 0.05 showing that the improvement in the IID is statistically significant and the null hypothesis that ‘eminectomy is ineffective for managing chronic closed lock’ can be rejected.

**Table 3:** Pre- and post-operative inter-incisal distance data from all three centres

	Hull	Dublin	Sunderland	Mean total
Mean pre-op IID (mm)	20.8	24.2	25.5	23
Mean post-op IID (mm)	37.7	33.9	38.3	37
Mean improvement IID	16.4	9.4	12.7	13

The complications experienced by the patients included in the cohort were recorded in all three centres. Surgical complications with regards to facial nerve weakness were temporary in six cases (4%), and permanent weakness was only found in three cases (2%). Temporary paraesthesia in the region of the auriculotemporal nerve was seen in 15 cases (9%), whilst three cases (2%) demonstrated altered sensation over the distribution of the Greater Auricular nerve. Permanent paraesthesia was present in seven cases (4%) over the distribution of the auriculotemporal nerve and two cases (1%) over the greater auricular nerve distribution. Three patients developed wound infection that was resolved with an oral antibiotic regime. In total, 108 patients out of 164 were discharged with a mean follow-up of 12 months (Table 4).

**Table 4:** Post-operative complications

Complication	Percentage of occurrence (%)
Facial nerve weakness	
Temporary	4
Permanent	2
Auriculotemporal nerve weakness	
Temporary	9
Permanent	4
Greater auricular nerve weakness	
Temporary	2
Permanent	1
Wound infection	2

## Discussion

The management of closed lock initially involves conservative medical management. Failed conservative medical treatment is usually followed by TMJ arthrocentesis. The procedure has been shown in the literature to be minimally invasive yet very effective, relieving symptoms in more than 80% of cases [7]. However, in this retrospective study, eminectomy has been shown as a safe and effective method that can be considered as a second-line surgical treatment option.

Recent studies by Sidebottom et al. 2013 show that open temporomandibular surgeries can benefit patients with previous failed arthroscopy to help manage their pain, restriction and locking, especially in Wilkes stage 3 [11]. The paper also highlighted that patients with Wilkes stage 5 had a higher risk of deterioration and poorer outcome after open temporomandibular joint surgery, and this should be considered an important predictor and must be highlighted in their consent [11, 12].

Articular eminectomy has long been used for the treatment of recurrent articular disc dislocation by allowing free movement of the condyles through removal of the obstruction. In 1984, Hall demonstrated the use of meniscoplasty combined with a rudimental eminectomy for the treatment of closed lock, experiencing some degree of success [13]. In 1994, Nitzan and Dolwick [14] postulated on the basis of 194 operated cases that adhesions caused by increased synovial viscosity and/or a hydraulic vacuum effect on the disc causing it to press against the slope of eminence can be alleviated by eminectomy [14]. Stassen et al. in a pilot study discussed that the procedure is safe and simple to use for treatment of a closed lock [8]. Eminectomy results in removal of the barrier to articular disc activity and therefore decompresses the intra-capsular compartment and creates a larger anterior recess in the superior joint space [15]. The term eminectomy has also been rephrased as eminoplasty and essentially involves the mechanical release by removal and recontouring of the bony obstruction of the eminence. It thereby facilitates improved mouth opening and subsequent resolution of clinical symptoms [7]. It is essential to point out that the procedure of surgical eminectomy/eminoplasty must ensure good bony clearance medially and smooth contouring of the articular fossa to allow the smooth translation of the joint. It is also essential to point out that some patients with an anteriorly displaced disc may have no symptoms despite MRI findings, and this can sometimes be explained occasionally by slow, natural resorption of anterior surface of condylar head or posterior aspect of the articular eminence to create the space to release the trapped disc-natural eminoplasty.

The contraindications for eminectomy are widely discussed in the literature [16]. There are a number of relative contraindications including chronic mandibular dislocations associated with a shallow articular eminence and radiographic evidence of a vascularised eminence [16]. A single, absolute contraindication to eminectomy has been noted where, in the presence of pneumatisation of the articular eminence, there is an increased risk of intracranial spread of inflammation along with an increased risk of temporal bone fracture [17]. The presence of pneumatisation can be identified through radiographic examination with both orthopantomogram (OPT) and computed tomography (CT) [18].

The visual analogue scale and Wilkes staging system are established and accepted forms of grading for internal derangement of the TMJ [11, 12]. The retrospective nature and geographical distribution of the study has led to these data being poorly recorded and its documentation would have further strongly supported this study. Current guidelines would be in conflict with this surgical procedure and would offer TMJ arthrocentesis as a first-line surgical treatment. The time frame over which the data have been collected and the large numbers make this study still an important contributor to the role of temporomandibular joint eminectomy as a second-line surgical option in selected cases, even in modern-day surgical management of refractory closed lock of TM joint.

This large, multi-centred retrospective study carried out at three different centres with consultant-led surgeries shows eminectomy to be a safe and reliable procedure in the treatment of closed lock. It shows statistically significant improvement in mouth opening (IID), where p was calculated to be < 0.05, with a mean IID of 37 mm at discharge from the clinic, 6 months post-operatively. There was complete resolution of a click in 58% of the cases and along with reduction in subjective pain symptoms in 56% cases leading to satisfactory discharge in 108 out of 164 cases.
